# Nanocomposite Based on Poly (para-phenylene)/Chemical Reduced Graphene Oxide as a Platform for Simultaneous Detection of Ascorbic Acid, Dopamine and Uric Acid

**DOI:** 10.3390/s20051256

**Published:** 2020-02-25

**Authors:** Zouhour Hsine, Salma Bizid, Rym Mlika, Hélène Sauriat-Dorizon, Ayoub Haj Said, Hafsa Korri-Youssoufi

**Affiliations:** 1 Université Paris-Saclay, CNRS, Institut de Chimie Moléculaire et des Matériaux d’Orsay (ICMMO), Equipe de Chimie Biorganique et Bioinorganique (ECBB), Bât 420, 2 Rue du Doyen Georges Poitou, 91400 Orsay, France; zouhourhsine1@gmail.com (Z.H.); helene.dorizon@universite-paris-saclay.fr (H.S.-D.); 2 Laboratory of Interfaces and Advanced Materials, Faculty of Science of Monastir, University of Monastir, Monastir 5019, Tunisia; bizid.salma@gmail.com (S.B.); mlikarym@yahoo.fr (R.M.); ahajsaid@gmail.com (A.H.S.)

**Keywords:** nanocomposite, reduced graphene oxide, poly(paraphenylene), ascorbic acid, dopamine, uric acid, urine

## Abstract

In this study, an efficient and simple designed nanohybrid created for individual and simultaneous detection of ascorbic acid (AA), dopamine (DA) and uric acid (UA). This nanohybrid is a combination of chemical reduced graphene oxide (CRGO) and redox poly(para-phenylene) (Fc-ac-PP) modified in a lateral position with ferrrocenyl group CRGO/Fc-ac-PPP. The CRGO/Fc-ac-PPP nanohybrid demonstrated a synergistic effect resulting in a large conductivity, surface area and catalytic properties provided by the redox attached ferrocene. Moreover, this nanocomposite is able to detect individually as well as simultaneously AA, DA and UA in a co-existence system with defined and separated redox peaks oxidation. The linear response ranges for AA, DA and UA, when detected simultaneously, are 0.1–10000 μM, 0.0001–1000 μM and 0.1–10000 μM, respectively, and the detection limits (S/N = 3) are 0.046 μM, 0.2 nM and 0.013 μM, respectively. The proposed sensor shown satisfactory results when applied to real spiked urine samples for measuring the abnormal high or lowconcentration of AA, DA and UA in vivo.

## 1. Introduction

The aim today in the domain of medical research and health care is the use of nanotechnology for the design of fast, efficient and quantitative diagnosis of biological substances to manage diseases and propose efficient therapy. In this context, the human body contains plenty of biological molecules in which monitoring their concentrations is crucial to disease screening. Dopamine (DA) is one of the important biological neurotransmitters in the mammalian central nervous system for message transfer [1]. The concentration of dopamine in the human body is low and it was found between 0.01–1 µM [2]. However, when it becomes lower than this value, it may result in neurological disorders, such as Parkinson’s disease and schizophrenia [3]. The low concentration of DA in the extracellular fluid for a sick patients and high in a healthy ones requires a sensitive method able to detect these compounds in a very large range of concentration. Many methods have been used for the determination of DA, such as fluorescence-quenching, mass spectrometry, and capillary electrophoresis. However most of these protocols are of high cost and time consuming [4,5] which limits their value in the case of rapid diagnosis. Electrochemical sensors are among the most convenient methods for the detection of dopamine due to their reliability for the rapid determination and quantitative determination of the analyte [6,7]. In addition, dopamine is an electroactive substance that makes electrochemical methods more suitable for its detection. However, various other electroactive compounds could be present in extracellular media and interfere in their detection using electrochemical methods. The two other molecules, AA and UA, coexist with a higher concentration (between 100 and 1000 times) than that of DA in biological fluids for healthy people [8]. These two molecules have redox signals at the potential range close to DA and make individual detection of dopamine difficult. Furthermore, these AA and UA, are markers for illness [9] when they are present at a high level or a very low level. In fact, the normal level of ascorbic acid (AA) is in the mM range and high level of AA can cause gastric irritation, and its metabolites can lead to renal issues, while the deficiency of AA is responsible for the illness scurvy [10,11]. In the case of uric acid (UA), the primary final product of purine metabolism to normal concentration in serum is 0.2 to 0.5 mM and the abnormal level of UA both in serum and urine is the symptom of a series of diseases including gout, hyperuricemia, Lesh-Nyhan syndrome, hypertension, urolithiasis, kidney and cardiovascular diseases [12]. Consequently, remarkable attention in biomedical and analytical investigations has been given to the design of rapid, sensitive, selective, and quantitative methods for direct detection of DA in the coexistence of AA and UA [13]. Therefore, the development of the electrochemical systems for simultaneous detection of AA, DA and UA with sensitive and selective method for their determination in in vivo analysis is the subject of many research projects [14,15]. This could be obtained by the modification of the electrode with the materials or nanomaterials improving the sensitivity and preventing overlapping. Various materials and nanomaterials have been studied for this purpose. Conducting polymers (CPs) with π-conjugated backbones have been widely used for enhancing the detection of AA, DA or UA thanks to their remarkable electronic properties [16,17]. Electroactive polymers integrating redox system demonstrate the catalytic effect that enhances the sensitivity toward AA, DA and UA detection [18,19]. Electrode-modified graphene have been also demonstrated for simultaneous detection of AA, DA and UA [20,21]. This is related to their electronic properties, surface area as well as their ability of chemical interaction with such substrates. In addition, it has been demonstrated that materials bearing negative charges and having an anionic form improve the selectivity of the biosensors, prevent non-specific interaction and enlarge the separation peaks between AA, DA and UA through electrostatic interaction [22,23]. The trends are now going for the development of nanocomposites that can bring various functionalities for improving electrochemical sensors. For example, reduced graphene oxide combined with conjugated polymers and anionic polymer polysulfonic acid have been demonstrated as efficient electrochemical sensing of neurotransmitters [24]. MoS2 nanospheres and polyaniline loaded on reduced graphene oxide were used for simultaneous detection of these neurotransmitters [25]. The detection limit in the micro-molar range is generally obtained in these sensors and difference in oxidation potential are less than 200 mV. 

In this work we aim to develop a sensor with enhanced electrochemical activity that improves sensitivity for electrochemical detection of small molecules without overlapping. A nanocomposite based on reduced graphene oxide (RGO) warped with poly(para-phenylene) modified with ferrocene and acidic group has been synthetized by simple methods and applied for simultaneous and individual electrochemical detection of AA, DA and UA. We demonstrate that the nanocomposite brings all the functionality for an efficient electrochemical sensor for various neurotransmitters in a large range and without overlapping. The RGO nanosheets provide a high surface area and conducting surface favoring electron transfer ability, the redox system brings electrocatalytic activity, and the anionic form prevents non-specific interaction and overlapping.

## 2. Materials and Methods

### 2.1. Reagents and Instrumentation

The polymer intituled “poly(para-phenylene)” modified with ferrocene and an acidic group in its backbone (Fc-ac-PPP) has been synthesized and characterized according to the previously reported procedure [26,27]. Gold electrode, graphene oxide (GO), dimethylformamide (DMF), ethanol, phosphate buffer solution (PBS tablets), AA, DA, UA, potassium ferrocyanide ([Fe(CN)_6_]^3−/4−^), ruthenium hexamine ([Ru(NH_3_)_6_]^2+/3+^) and urine samples were purchased from Sigma Aldrich (France). The interfering substances used in this study were also purchased from Sigma Aldrich. Prior the measurements, the prepared PBS was purified by 0.22 µm syringe filters. All solutions have been prepared using deionized water produced by milli-Q water system. All solutions have been freshly prepared each day. The instrumentations are detailed in supplementary information.

### 2.2. Synthesis of Chemical Reduced Graphene Oxide (CRGO)

The reduction of the graphene oxide (GO) to obtain chemical reduced graphene oxide (CRGO) has been made by the optimized chemical approach according to the method described in the literature [28]. The reaction has been performed in DMF at 80 °C during 1h. After several centrifugation and washing steps, the CRGO powder was obtained. 

### 2.3. Nanocomposite Formation CRGO/Fc-ac-PPP 

The nanocomposite has been prepared by mixing Fc-ac-PPP polymer with CRGO in DMF followed by ultrasonication during 30 min to allow the attachment of the polymer on the surface of graphene nanosheets. To optimize the nanocomposite properties various ratio of CRGO/Fc-ac-PPP have been studied in order to obtain the nanocomposite with high electroactivity and electronic properties. Therefore, hybrids with different mass ratio (0.1:5, 0.5:5, 1:5, and 2:5) have been prepared. 

**Scheme 1:**
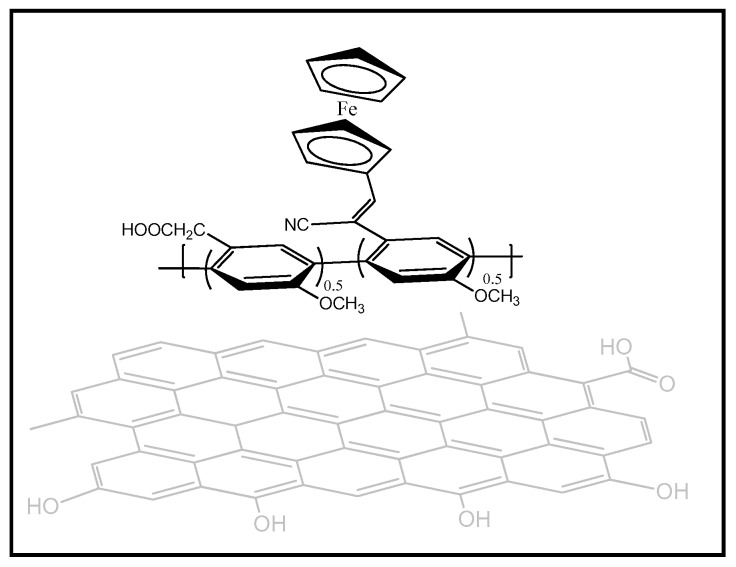
Chemical structure of the nanocomposite.

For the sensor formation, the gold surface, after polishing steps, has been dropped with the nanocomposite CRGO/Fc-ac-PPP (Scheme 1) suspension followed by a drying step under oven at 80 °C for 45 min. The membranes based on the nanocomposite CRGO/Fc-ac-PPP with different mass ratio were characterized using cyclic voltammetry (CV) and electrochemical impedance spectroscopy (EIS) to underline the best ratio for sensor application.

## 3. Results and Discussions

### 3.1. Characterization of CRGO/Fc-ac-PPP Nanocomposite

The structure of CRGO was analyzed by X-ray photoelectron spectroscopy (XPS) and Fourier-transform infrared spectroscopy (FT-IR), CV and EIS measurements to underline the efficiency of reduction method. FT-IR spectra of GO show various oxygen functionalities after reduction, and various bands related to oxyganed species in GO disappear in the FT-IR spectrum of CRGO which confirm the deoxygenation of GO after reduction (Supplementary Materials Figure S1A). The same behavior is observed for XPS analysis. Reduction of GO removes the oxygen band as observed in XPS spectra (Supplementary Materials Figure S1B,C) where the oxygen band was removed from carbon region for CRGO. Electrochemical characterizations through CV and ESI show also an improvement in electron transfer ability demonstrating the restoration of the aromatic system (Supplementary Materials Figure S2).

The nanocomposite formation CRGO/Fc-ac-PPP has been obtained by mixing the CRGO and the soluble polymer in DMF. The association of the two materials can be obtained through the π-π staking interaction as demonstrated previously in the case of a carbon nanotube [29]. A scanning electron microscope (SEM) image was obtained of the surface modified with RGO and polymer, and the nanocomposite show clearly the warping of graphene nanosheets with the polymer (Supplementary Materials Figure S3).

The study of ratio optimization was performed following redox signal of ferrocene in the nanocomposite by cyclic voltammery measurement of the redox ferrocene attached to the polymer and the electrical behavior through ESI (Supplementary Materials Figure S4). Such analysis demonstrates that the optimal signal of redox ferrocene in the nanocomposite is obtained for 1:5 ratio of CRGO/Fc-ac-PPP where high redox signal intensity was obtained (Supplementary Materials Figure S4A). ESI shows also a decrease of the Nyquist semicircle related to charge transfer resistance (Supplementary Materials Figure S4B) demonstrating the efficient electron transfer ability of redox ferrocene in the membrane of nanocomposite.

### 3.2. Electrochemical Characterizations

**Figure 1:**
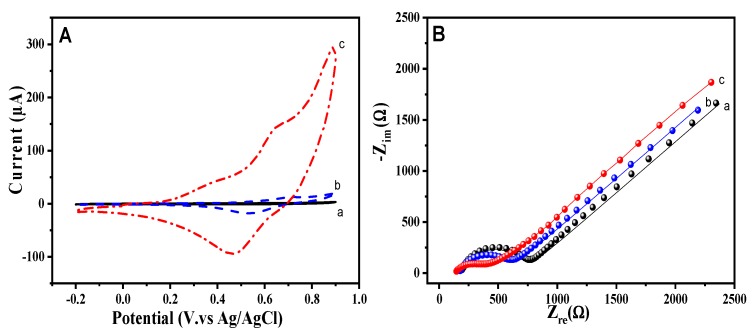
(**A**) CVs of various electrodes in 10 mM PBS solution scan rate 50 mv/s gold modified, Au (a), Au/ Fc-ac-PPP (b), Au/ CRGO/Fc-ac-PPP (c); (**B**) ESI measured in PBS solution with amplitude 10mV and frequency range 100mHz to 100KHz; Au (a), Au/ Fc-ac-PPP (b), Au/ CRGO/Fc-ac-PPP (c).

When comparing the redox properties of the electrode modified with polymers and nanocomposite with CV (Figure 1A), the redox signal of ferrocene attached on the polymer in nanocomposite (Figure 1A, curve c) shows higher intensity than in the case of the polymer (Figure 1A, curve b). This is provided by the surface area of graphene nanosheets ESI recorded with the same surface (Figure 1B) demonstrates also a decrease of diameter of a semi-circle of the Nyquist plot for the electrode modified with nanocomposite ( Figure 1B, curve c) compared to polymer (Figure 1B, curve b) corresponding to a decrease of charge transfer resistance. This behavior demonstrates the efficient interaction of graphene and polymer thanks to various possibilities of interaction obtained with graphene nanosheets. This could be through the π–π staking with aromatic ring or the ionic interaction from the carboxylic side chain attached to the redox polymer.

**Figure 2:**
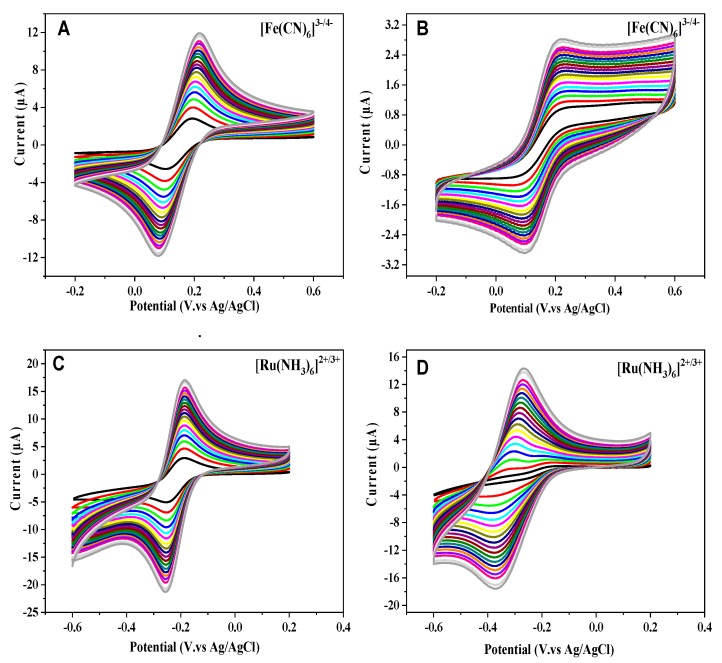
CVs curve of the nanocomposite CRGO/Fc-ac-PPP and Fc-ac-PPP modified gold electrodes analyzed in solution of 10mM [Fe(CN)6]^3−/4−^ (curve **A**,**B**) and [Ru(NH3)]^2+/3+^ (**C**,**D**), with various scan rate from 5 to 1000 mV/s.

Electrochemical performance of the nanocomposite CRGO/Fc-ac-PPP have been performed using two well-known redox systems, [Fe(CN)_6_]^3−/4−^ surface-sensitive redox marker “inner-sphere” and [Ru(NH_3_)_6_]^2+/3+^, outer-sphere redox marker, which can provide information about the electronic properties of the nanocomposite and the density of electronic states (DOS) [30]. The kinetics of heterogeneous electron transfer (*k*s) of the redox markers were calculated within the polymer and nanocomposite. Nicholson method based on the variation of the peak potential versus scan rate was used to calculate the *k*s using the dimensionless parameter *ψ* plotted versus scan rate (*ν* − 1/2) [31] (Supplementary Materials Figure S5). Figure 2A,B show the CVs at different scan from 50 mVs^−1^ to 1000 mVs^−1^ for [Fe(CN)_6_]^3−/4−^ at surface modified with CRGO/Fc-ac-PPP and compared to Fc-ac-PPP. The variation of anodic and cathodic peak potentials (△EP) shows a decrease from 163.31 mV for the polymer to 87.39 mV for the nanocomposite, which highlights that the reversibility of the redox system is improved in the case of the nanocomposite. This behavior demonstrates the fast diffusion of the redox marker to the nanocomposite provided by graphene nanosheets. The calculated *ks* found to be 5.4 × 10^−3^ cm s^−1^ and 6.4 × 10^−3^ cm s^−1^ for surfaces modified with Fc-ac-PPP and CRGO/Fc-ac-PPP, respectively.

In the case of CVs obtained with [Ru(NH_3_)_6_]^2+/3+^, the same behavior has been obtained where ΔEP decreases from 133 to 62 mV in the case of the nanocomposite (Figure 2C,D). This indicates the enhance of electron transfer ability due to the increase of the conductivity of the nanocomposite. Furthermore, the calculated *ks* with the same method shows an improvement in the case of the nanocomposite with values calculated from 0.035 cm s^−1^ to 0.075 cm s^−1^. This behavior is in agreement with an improvement of the density of electronic states (DOS) of CRGO/Fc-ac-PPP thanks to the electronic effect of the conjugated polymer.

These results confirm that the association of Fc-ac-PPP to CRGO provides a nanomaterial with a large surface area and a high conductivity where the electron transfer ability to the redox system can lead to a sensitive surface for the electrochemical detection of AA, DA, and UA. 

### 3.3. Electrochemical Activities of AA, DA and UA on Modified Surfaces

**Figure 3:**
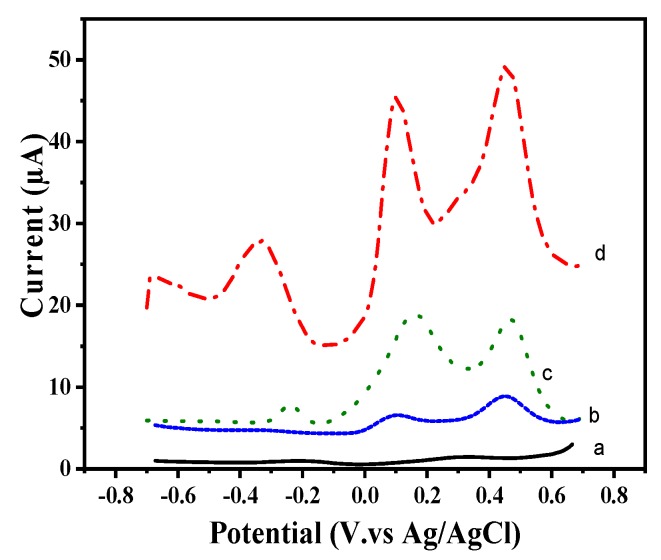
DPV responses of the mixture of 10^−4^ M ascorbic acid (AA); 10^−5^ M dopamine (DA); 10^−4^ M uric acid (UA) in 0.1 M phosphate buffered saline (PBS) (pH = 7) on (a) bare Au (b)CRGO (c) Fc-ac-PPP and (d) CRGO/Fc-ac-PPP.

To evaluate the electrochemical activity of the nanocomposite CRGO/Fc-ac-PPP toward AA, DA and UA, individual and simultaneous detection of these analytes have been investigated at the gold electrode, non-modified (Au), modified with the polymer (Au/Fc-ac-PPP) and modified with the nanocomposite (Au/CRGO/Fc-ac-PPP). Differential pulse voltammetry (DPVs) have been performed with the 3 compounds in mixture of 10^−4^ M UA and AA and 10^−5^ M DA (Figure 3) and individually detection (Supplementary Materials Figure S6). The nanocomposite CRGO/Fc-ac-PPP depicts a very well-defined redox signal with intense peak current for each analyte compared with bare Au and Au/Fc-ac-PPP. Moreover, the peak potential of the individual detection of AA, DA or UA at Au/CRGO/Fc-ac-PPP shifts to a lower potential range compared to the other electrodes. This behavior proves that the kinetics of electron transfer of these analytes has been faster on the nanocomposite than the other electrodes [32]. In addition, the oxidation peaks are separated well compared to the other electrodes. ∆Ep has been obtained 455 mV for peak separation AA-DA and 354 mV for DA-UA.

Individual detection shows the same behavior (Supplementary Materials Figure S6), the nanocomposite Au/CRGO/Fc-ac-PPP exhibits also the highest peak current intensity of AA, DA and UA oxidation compared to the other electrodes.

The high performance of Au/CRGO/Fc-ac-PPP compared to Au/ Fc-ac-PPP could be related to high surface provided by the RGO as well as the electronic properties of such composite with an improvement of electron transfer ability. 

### 3.4. Analytical Performance of Au/CRGO/Fc-ac-PPP Sensor

**Figure 4:**
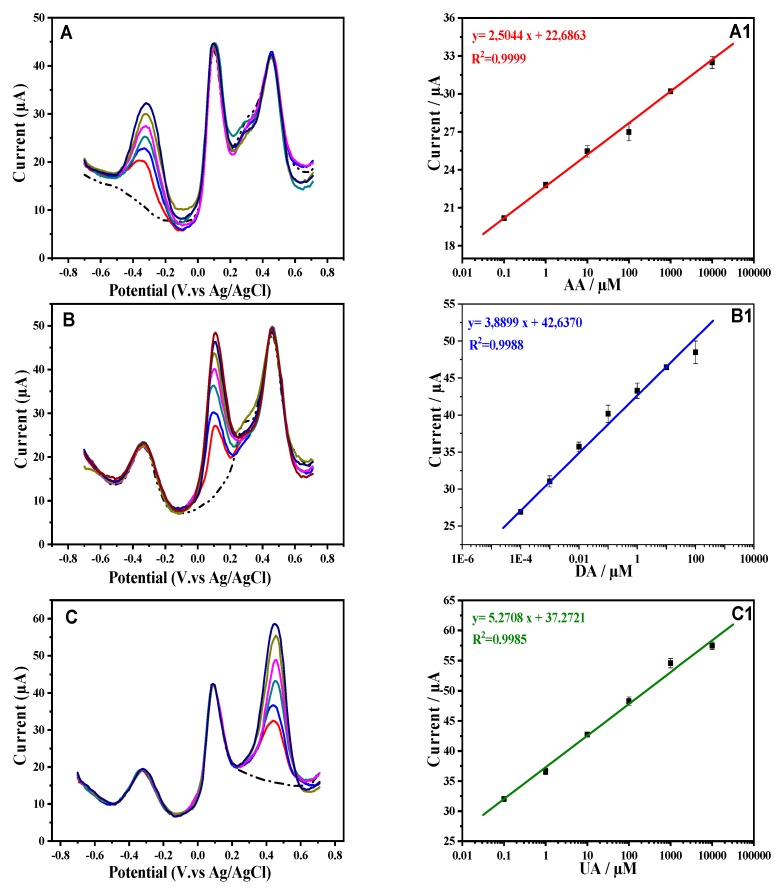
DPV curves of CRGO/Fc-ac-PPP in 0.1 M PBS with varying concentrations of (**A**) AA: 10^−7^ M; 10^−6^ M; 10^−5^ M; 10^−4^ M; 10^−3^ M; 10^−2^ M (**B**) DA: 10^−10^ M; 10^−9^ M; 10^−8^ M; 10^−7^ M; 10^−6^ M; 10^−5^ M; 10^−4^ M and (**C**) UA: 10^−7^ M; 10^−6^ M; 10^−5^ M; 10^−4^ M; 10^−3^ M; 10^−2^ M. The corresponding plot of (**A1**) Ipa vs. C_AA_ (**B1**) Ipa vs. C_DA_ and (**C1**) Ipa vs. C_UA_.

To underline the detection signal within the concentration of each compound in the presence of the other compounds, where the concentration of the target biomolecule has been changed, while the concentrations of the other two biomolecules were kept constant, were analyzed by DPV with Au/CRGO/Fc-ac-PPP. Figure 4A−C shows the DPV curve of the mixture with increasing concentration of one biomolecule. The electrochemical response shows an increase of redox signal within the concentration while the other signals remain constant. Based on the variation of the intensity of peak current related to added concentration of biomolecules, calibration curves of AA, DA and UA were plotted versus logarithm scale of the concentration with correlation coefficients of 0.99 (Figure 4(A1−C1)). Detection limits (LOD) were calculated based on signal to noise ratio 3 at 4.6 × 10^−8^ M, 2.8 × 10^−10^ M, 1.3 × 10^−8^ M for AA, DA and UA respectively. These results demonstrate that the nanocomposite CRGO/Fc-ac-PPP can be applied to individual as well as for simultaneous detection of AA, DA, and UA. Compared with some modified electrode published in the last few years (Table 1), Au/CRGO/Fc-ac-PPP sensor presents the lowest LOD for AA, DA and UA and the wide range for their detection and high peak separation. DPV as well as chronoamperometry could be used to follow the presence of these compounds. In addition, the fabrication process is easy compared to other presented sensors. The power of this sensor, beside its analytical performance, resides in the fact that it is fabricated using organic molecules without any metallic nanoparticles which is eco-compatible. 

**Figure 5:**
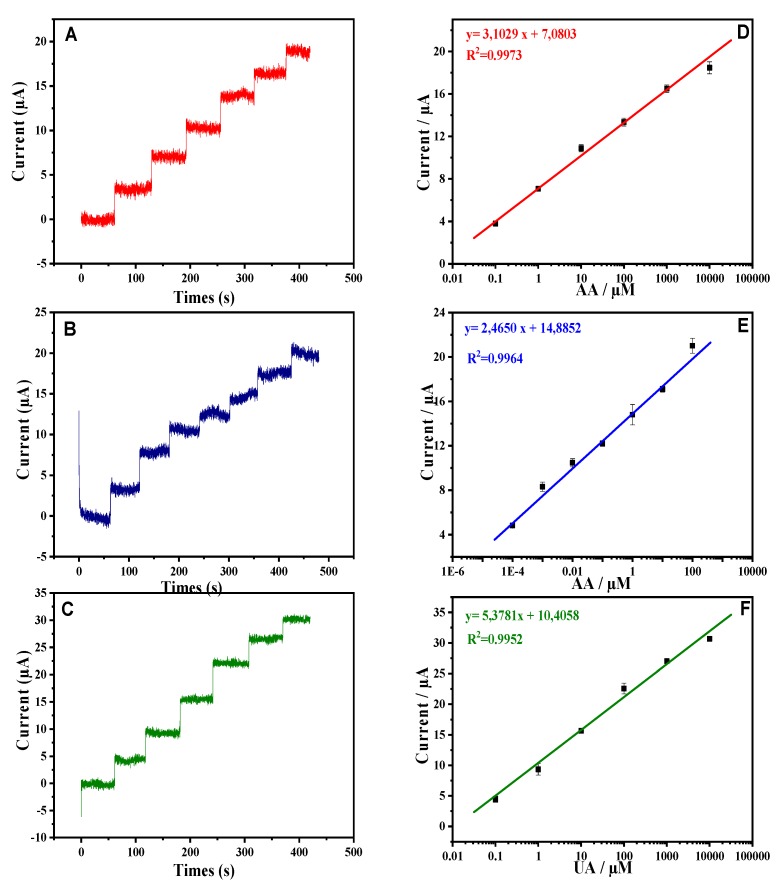
Amperometric responses of the CRGO/Fc-ac-PPP electrode after the subsequent addition of (**A**) [10^−7^ M; 10^−6^ M; 10^−5^ M; 10^−4^ M; 10^−3^ M; 10^−2^ M] of AA at applied potential of −344.50 mV, (**B**) [10^−10^ M; 10^−9^ M; 10^−8^ M; 10^−7^ M; 10^−6^ M; 10^−5^ M] of DA at 101.21 mV and (**C**) [10^−7^ M; 10^−6^ M; 10^−5^ M; 10^−4^ M; 10^−3^ M; 10^−2^ M] of UA at 460.51 mV solution in a 010mM PBS. The corresponding calibration curves of AA (**D**), DA (**E**), and UA (**F**).

To evaluate the analytical performance of the Au/CRGO/Fc-ac-PPP electrode, chronoamperometric measurements have been devoted as this method is easy to use. The response was obtained in a few seconds and can be employed for the individual detection of AA, DA, UA by selecting working potentials of each biomolecule. These measurements have been made in stirred PBS and the concentration of each target molecule was added with applied potential of −344.50 mV, 101.21 mV, and 460.51 mV for the detection of AA, DA, and UA, respectively. Figure 5 shows that current increases after each addition of AA, DA and UA. The calculated electrochemical sensor response time for AA, DA and UA were 0.36 s, 1.03 s and 0.32 s respectively which demonstrates the fast response of the Au/CRGO/Fc-ac-PPP and its sensitivity to small changes in the concentration of the target molecule. Calibration curves corresponding to the variation of steady state current with log of concentration of AA, DA, and UA shows a linear variation. The detection limit for AA, DA and UA were calculated to be 2.3 × 10^−8^ M, 1.3 × 10^−11^ M and 1.2 × 10^−8^ M respectively based on S/N = 3 which is the same as is observed in DPV measurement. Comparing the analytical performances of sensors CRGO/Fc-ac-PPP with previous reported sensors (Table 1), various properties are improved: peak potentials separation, range of detection, LOD for the three species and simultaneous determination of AA, DA, and UA without any overlapping. 

### 3.5. Interferences, Stability and Reproducibility

**Figure 6:**
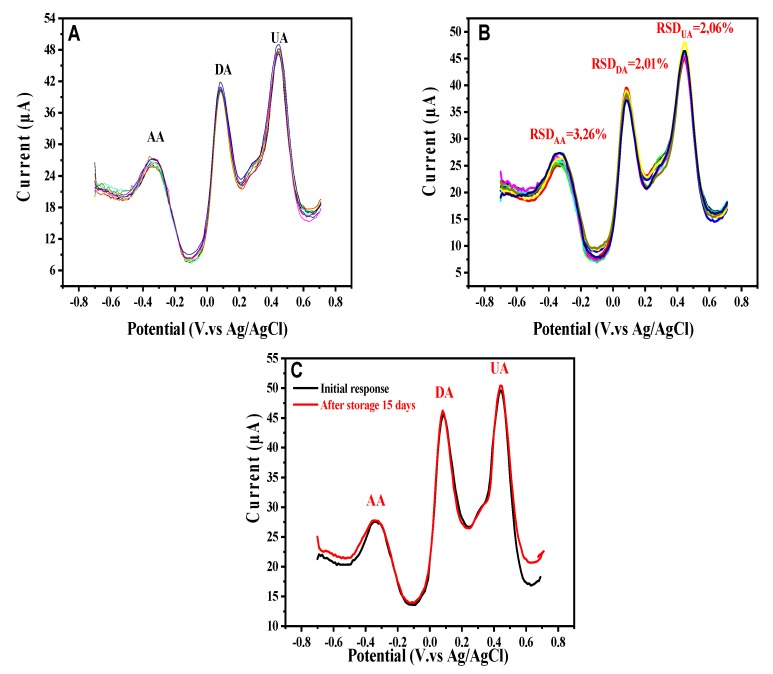
(**A**) DPV of Au/CRGO/Fc-ac-PPP in 0.1 M PBS containing 10^−4^ M AA and UA and 10^−5^ M in the presence of glycine (red), serotin (green), glutamic acid (blue), glucose (light blue), citric acid (pink), KCl (brown); KNO_3_ (dark blue), Na_2_SO_4_ (purple). (**B**) Reproducibility and (**C**) stability studies at Au/CRGO/Fc-ac-PPP in 0.1 M PBS containing 10^−4^ M AA and UA and 10^−5^ M DA.

The effect of interferences on the Au/CRGO/Fc-ac-PPP sensor response has been analyzed in the presence of several co-existing substances in biological fluids (glycine, serotonin, glutamic acid, glucose, citric acid, NaCl, KCl, KNO_3_, Na_2_SO_4_). Figure 6A depicts the DPV curves of Au/CRGO/Fc-ac-PPP in PBS electrolyte containing 10^−4^ M AA and UA and 10^−5^ M DA after the addition of 1 mM of each interference substances. The addition of interference molecules does not affect the current of redox system of AA, DA and UA which confirms the selectivity of the sensor. 

The reproducibility of the sensor has been evaluated by nine equally prepared electrodes and their analysis by DPV in the mixture of 10^−4^ M AA, UA and 10^−5^ M DA (Figure 6B). The oxidation peak potentials and currents of the three species were almost the same. The relative standard deviation (RSD) was calculated at 3.2%, 2% and 2% for AA, DA, and UA, respectively. 

The storage stability of the Au/CRGO/Fc-ac-PPP electrode has also been investigated. The sensor has been kept at ambient temperature for 15 days and then used for the simultaneous detection of AA, DA and UA (Figure 6C). AA, DA, and UA oxidation retained 99.4%, 98.3%, and 98.4% of its initial current response which proves the stability of the sensor due to the stability of the nanocomposite.

### 3.6. Determination of DA in Biological Fluids

In order to confirm the validity of the sensor for real applications, simultaneous detection of AA, DA, UA in a urine sample has been performed using a standard addition method. The urine sample free of neurotransmitters was diluted 10 times with PBS to prevent matrix effect. Then, various urine samples have been mixed with various concentrations of these neurotransmitters to test the simultaneous detection where various levels coexist. The urine was spiked with various concentrations of AA, DA and UA and the DPV measurement was performed in the same potential region used in PBS. Various samples were tested with various levels, urine⋕1 and urine⋕2 are for a normal level of AA and DA and UA, urine⋕3 and ⋕4 for disorders in AA, DA and UA. The redox responses obtained in urine were at the same potential as PBS solution. The recovery has been calculated (Table 2) and showed values from 110% to 90% depending of the concentration of neurotransmitters. RSD of less than 3% was calculated which proves the accuracy of the measurement. 

Hence these three analytes can be easily determined at Au/CRGO/Fc-ac-PPP sensor without any interference and can be applied to diagnose patients suffering from carcinoid syndrome, Parkinson and hypertension [39]. These results demonstrate that the proposed sensor based on CRGO/Fc-ac-PPP nanocomposite has a potential for its application in diagnosis.

## 4. Conclusions 

In this work, a nanocomposite formed with reduced graphene oxide and Fc-ac-PPP polymer has been created and analyzed as a sensor for individual and simultaneous electrochemical detection of AA, DA and UA. The characterization of the nanocomposite by CV, EIS and FTIR have demonstrated the good distribution, the warping of the polymer with RGO through π-π staking interaction which undergoes the improvement of the surface area, conductivity and electro-catalytic activity. In addition, the nanocomposite showed faster kinetic charge transfer than Fc-ac-PPP using an inner and outer-sphere redox probe. The gold surface modified with nanocomposite allowed successful detection of AA, DA and UA in a ternary mixture with well-separated voltammetry peaks. This new nanocomposite can either detect single species separately or determine the concentration of one analyte in the presence of the two other analytes without any overlapping. The sensor can detect various concentration ranges in urine samples which makes it a powerful platform for medical diagnosis. The easy process of sensor fabrication made by eco-compatible organic materials and providing high analytical performances towards electroactive biomolecules could be generalized to the detection of other molecules of interest.

## Figures and Tables

**Table 1:** Analytical parameters for the simultaneous determination of ascorbic acid (AA), dopamine (DA), uric acid (UA) based on DPV (*) i-t measurements using different modified electrode PMES (poly(2-(*N*-morpholine) ethane sulfonic acid); (GCE) glassy carbon electrode; (GS) graphene sheet; (GS) chitosan, (PEDOT) poly(3,4-ethylenedioxythiophene); (PANI) polyaniline; (NC@GC) hierarchical core-shell metal-organic framework; (h-ATS) mesoporous silver-doped TiO_2_-SnO_2_ nanocomposite.

Modified Electrode	Dynamic Range (µM)			LOD (µM)			ΔEP (mV)		**Ref**
Analyte	AA	DA	UA	AA	DA	UA	AA-DA	DA-UA	
PMES/RGO/GCE	30−100	0.05−100	0.1−100	0.43	0.0062	0.056	-	-	[33]
GCE/GS/CS	-	1−700	1−800	-	0.14	0.17	-	-	[34]
GCE/RGO/ZnO	50−2350	1−70	3−330	3.77	0.33	1.08	236	132	[35]
GCE/PEDOT/PANI	10^−4^−10^2^	30−1000	0.7−100	24.2	4.58	2.23	216	320	[36]
GO/PEDOT/GCE	100−1000	6.0−200	40−240	20	2	10	97	129	[37]
ITO/g-C3N4/NC@GC/h-ATS	0.1−200	2.5−100	2.5−500	0.02	0.01	0.06	236	204	[38]
GONRs/PETDOT/PSS	250−1500*	0.5−800	0.5−1200	250	0.5	0.5	220.5	115.1	[21]
	*0.05−16.55	0.05−16.55	0.05−1655	0.041	0.030	0.011			
Au/CRGO/Fc-ac-PPP	0.1−10^4^	10^−4^−10^3^	0.1−10^4^	0.046	2.8×10^−5^	0.013	445.7	359.30	This work
	*0.1−10^4^	10^−4^−10^3^	0.1-10^3^	0.022	1.2×10^−5^	0.012			This work

**Table 2:** Simultaneous determination of AA, DA and UA in urine sample (n = 3).

Sample	Analyte	Added (µM)	Found (µM)	Recovery %	RSD %
**Urine** ⋕ **1**	**AA**	1000	1010	101	0.4
	**DA**	10	11.1	111	2.1
	**UA**	1000	1020	102	0.5
**Urine** ⋕ **2**	**AA**	100	85	85	0.9
	**DA**	0.1	0.1	100	1.5
	**UA**	100	100	100	0.9
**Urine** ⋕ **3**	**AA**	10	10.5	105	0.4
	**DA**	0.01	0.0097	97	3.4
	**UA**	10	10.5	105	0.2
**Urine** ⋕ **4**	**AA**	0.1	0.095	95	2.4
	**DA**	0.001	0.001	100	2.7
	**UA**	0.1	0.11	110	0.6

## Supplementary Materials

The following are available online at https://www.mdpi.com/1424-8220/20/5/1256/s1.
