# Supplementary material for: Nanocomposite Based on Poly (para-phenylene)/Chemical Reduced Graphene Oxide as a Platform for Simultaneous Detection of Ascorbic Acid, Dopamine and Uric Acid
Source: Sensors (Basel). 2020 Feb 25;20(5):1256. doi: 10.3390/s20051256 (PMC7085538; doi:10.3390/s20051256)
Supplement: Supplementary file 1 [file sensors-20-01256-s001.pdf]

SUPPLEMENTARY MATERIALS INFORMATION

# Nanocomposite based on Poly (para-phenylene) /Chemical Reduced Graphene Oxide as a Platform for Simultaneous Detection of Ascorbic Acid, Dopamine and Uric Acid

## SI.1. Material and Methods

### *SI.1.1 Reagents*

The polymer intituled "poly(para-phenylene)" modified with ferrocene and acidic group in itsbackbone (Fc-ac-PPP) has been synthesized and characterized according to the previously reported procedure [1-2].

Gold electrode, graphene oxide (GO), dimethylformamide (DMF), ethanol, phosphate buffer solution (PBS tablets), ascorbic acid (AA), dopamine (DA), uric acid (UA) , potassium ferrocyanide ($[Fe(CN)_6]^{3-/4-}$ ), ruthenium ($[Ru(NH_3)_6{}^{2+/3+}]$) and urine samples were purchased from Sigma Aldrich (France). The interferent substances used in this study were also purchased from Sigma Aldrich. Prior to the measurements, the prepared PBS was purified by 0.22 μm syringe filters. All solutions were prepared using deionized water produced by milli-Q water system. All solutions were freshly prepared each day.

### *SI.1.2 Instrumentations*

**Cyclic voltammetry (CV) and electrochemical impedance spectroscopy (EIS)** measurement were recorded using a Metrohm Autolab PGstat 12 controlled by Nova® software.

**Differential pulse voltammetry (DPV) and chronoamperometry (CA)** were recorded using Metrom Autolab supported by EC-Lab software.

The electrochemical measurements were carried out in an electrochemical cell involving three electrodes purchased from BASi: a gold disc (surface 2.01 $10^{-2}$ $cm^2$ ) as a working electrode, a platinum wire as a counter electrode and an Ag/AgCl as a reference electrode.

**Fourier transform infrared spectroscopy (FT-IR)** characterizations were obtained using a Bruker IFS66 FT-IR spectrometer (Bruker, Germany) equipped with a mercury-cadmium-telluride (MCT) detector and an attenuated total reflectance (ATR) germanium crystal.

**Scanning electron microscope (SEM)** images were performed with a Hitachi scanning electronic microscope (SEM 4800).

**X–ray photoelectron spectroscopy (XPS)** measurements were performed by ThermoFisher using a monochromatic Al K$\alpha$ X–ray source. Digital acquisition was achieved with a computerized Advantage system.

## SI.2. Results and Discussion

### *SI.2.1. Characterization of the Chemically Reduced Graphene Oxide (CRGO)*

For the reduction of GO, we followed the optimized reduction strategy given by Martin Kühnel et al [1] who demonstrated the synthesis of a high and pure quality of RGO. To make sure of all this, GO and CRGO were characterized using cyclic voltammetry (CV), Electrochemical impedance spectroscopy (EIS) and X-ray photoelectron spectroscopy (XPS).

**Figures S1B and S1C** Show the C1s XPS spectrum of GO before and after the reduction. The C1s XPS spectrum of GO **(Figure S1B)** can be divided into five different peaks; the non-oxygenated

ring C=C at 284.30 eV and C-C at 285.04. The other three oxygenated C atoms are observed at 285.70 ev, 287.15 ev and 288.70 ev which correspond to C-OH, C=OH, and COOH respectively. The proof of the good formation of CRGO is that the C1s XPS spectrum of CRGO **(Figure S1C)** showed the same functional groups of GO, but with dramatically decreased intensities and shifted peak positions of the oxygenated groups. On the other hand, an increase of C=C bond peak was observed which demonstrated the restoration of $sp^2$ networks on the basal plane of CRGO. The XPS analysis validates that most of the oxygen-containing functional groups are removed, resulting in an increase of $sp^2$ networks on the basal plane of CRGO [2]. In addition, this reduction can also be clearly confirmed by the colour change of the aqueous dispersion solutions of GO and CRGO from yellow to black (Figure S1D).

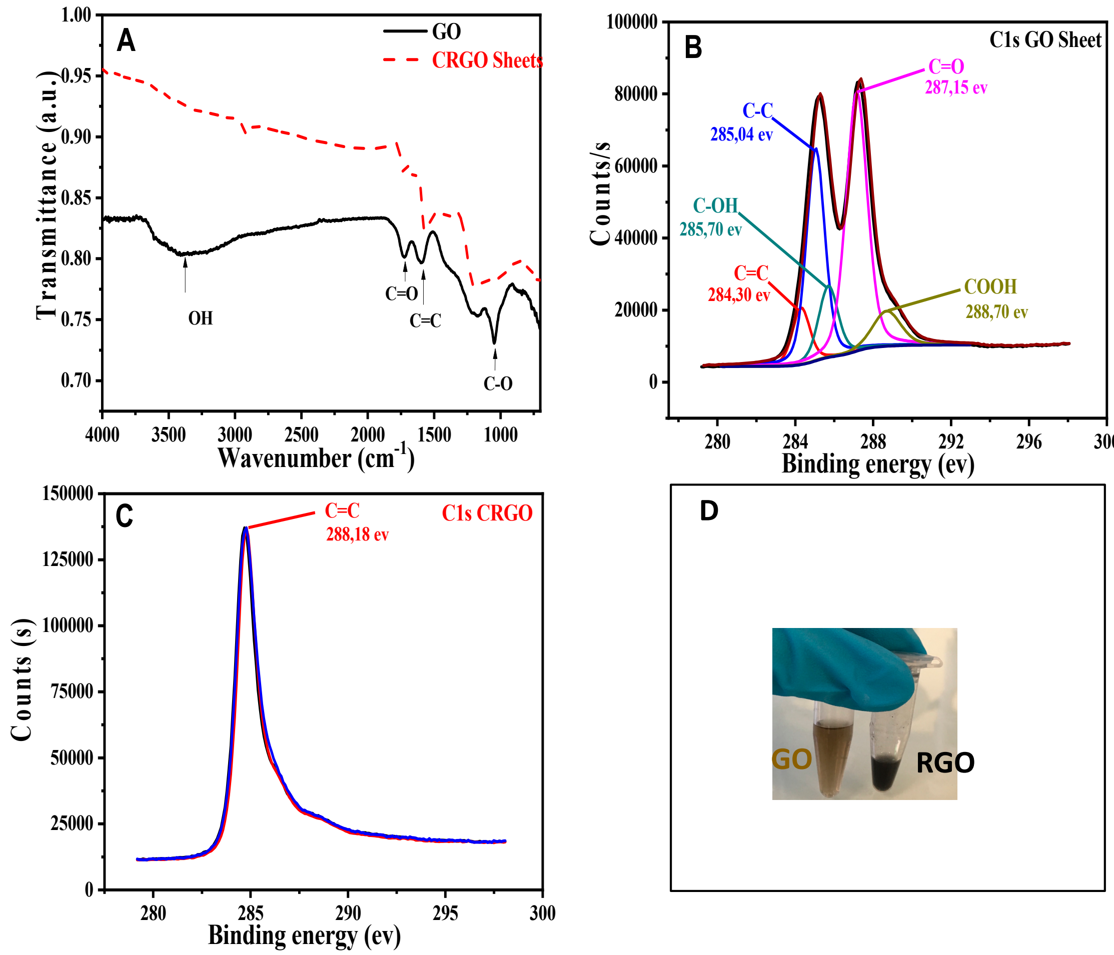


**Figure S1.** FT-IR spectra (A) Fourier transform infrared spectroscopy (FTIR) spectra of GO and CRGO. X-ray photoelectron spectroscopy (XPS) spectra in carbon region of (B) GO and (C) CRGO. (D) Photographs of the GO solution before and after reduction.

The Electrochemical characterization of the Chemical Reduced graphene oxide (RGO) has been made by cyclic voltammograms (CV) and Electrochemical impedance spectroscopy (EIS).The CV of 10 mM $[Fe(CN)_6]^{3-/4-}$ on GO and CRGO modified gold electrodes were recorded in 0.1M of KCl at a scan rate of 50 $mVs^{-1}$ **(Figure S2 A)**. A pair of redox peaks has been observed for both modified electrodes corresponding to the redox reaction between the $[Fe(CN)_6]^{3-/4-}$ redox couple. After the chemical reduction of GO, the redox peak current increases distinctively and the ΔEp have decreased from 391 mV for GO to 163.31 mV for CRGO indicating that CRGO has accelerated electron transfer between the electrochemical probe and the Au electrode. In fact, the reduction of the oxygenated groups from the surface of GO leads to the decrease of the repulsion between these groups and the negatively charged species of $[Fe(CN)_6]^{3-/4-}$, and consequently the enhancement of the signal and charge transfer that we observed on the Au/CRGO electrode. These results may be attributed to the increase in the electrical conductivity after reduction, probably due to the restoration of the π-conjugated system induced by the deoxygenation [3].

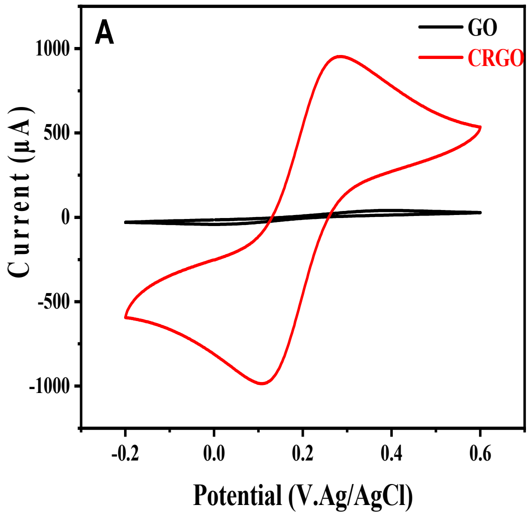


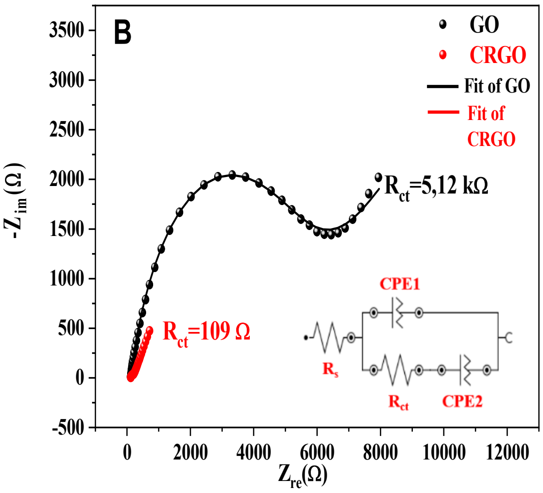


**Figure S2.** (A) Cyclic voltammetry (CV) of GO and CRGO in the solution of 10mM $[Fe(CN)_6]^{3-/4-}$ in 0.1M KCL at a scan rate of 50 mVs $^{-1}$. (B) Electrochemical impedance spectroscopy (EIS) of Au modified with GO and RGO analyzed in solution of 10mM $[Fe(CN)_6]^{3-/4-}$ in 0.1M KCl with amplitude of 10mV and frequency range from 0.1Hz to 100KHz.

To better understand the superior electrochemical performance of CRGO which is coming from the reduction of GO, EIS of the GO, and CRGO was carried out in potassium ferri/ferro-cyanide solutions (10 mM $[Fe(CN)_6]^{3-/4-}$ , 0.1 M KCL) **(Figure S2 B)**.The experimental results were fitted with the equivalent circuit presented in **Inset Figure S2 B** which gives the best fit with a low total error ($\chi^2$). The equivalent circuit formed by the electrolyte resistance (*Rs*), the charge transfer resistance at the membrane/electrode interface (*Rct*), the constant phase element (*CPE1*) represents the double layer capacity and the constant phase element (*CPE2*) is related to the space charge capacity in the membrane. Based on the EIS data fitting, the Au/CRGO electrode exhibits a small value of $R_{ct}$ (109 Ω) compared to the value of the Au/GO (5.12 kΩ). This result proves that the CRGO membrane enhanced the charge transfer of the Au electrode which proves the good reduction of GO.

*SI.2.2. Formation and Optimization of the CRGO/Fc-ac-PPP*

Scanning electron microscope (SEMs) image of the nanocomposite

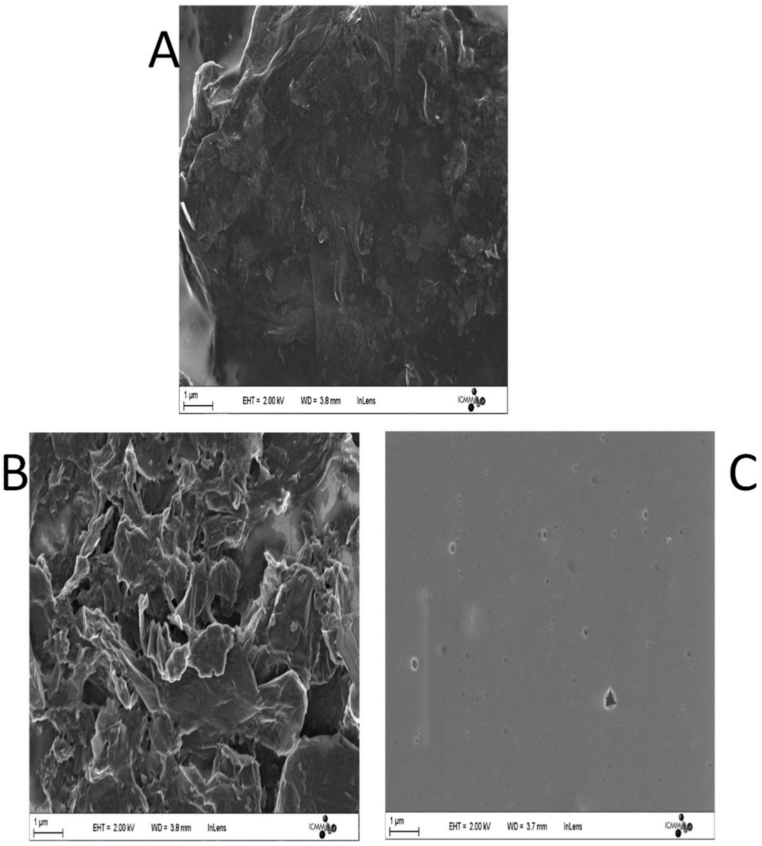


**Figure S3.** Scanning electron miscroscope (SEM) images of (A) nanocomposite CRGO/Fc-ac-PPP compared to (B) CRGO and (C) Fc-ac-PPP.

CRGO/Fc-ac-PPP hybrids solutions with different mass ratio (0.1:5, 0.5:5, 1:5, and 2:5) were prepared and then used for the electrochemical measurement by CV and EIS in PBS (0.1 M KCL, pH=7. For EIS measure, the equilibrium potential of each mass ratio was taken from CV **(Figure S4A)**. **Figure S4B** represent the diagrams of the different mass ratio of the nanocomposite CRGO/Fc-ac-PPP. These EIS curves were fitted by the equivalent circuit, presented in **inset Figure S4B**. The equivalent circuit Warburg s impedance (Zw) corresponds to the diffusion of ions through the bulk of the membrane for the redox reaction (*Rs*); the charge transfer resistance at the membrane/electrode interface (*Rct*); the constant phase element (*CPE*) represents the double layer capacity and the warburg s impedance (Zw) corresponds to the diffusion of ions through the bulk of the membrane. Based on the equivalent circuit fitting, the $R_{ct}$ value for the mass ratio 0.1:5, 0.5:5, 1:5 and 2:5 were found to be 855Ω, 533Ω, 242Ω and 677Ω ,respectively. Hence, the nanocomposite with mass ratio 1:5 represents the lower $R_{ct}$, consequently the higher conductivity and faster charge transfer. Through these characterizations, the nanocomposite with the mass ratio 1:5 will be chosen as the optimized nanocomposite for the following work.

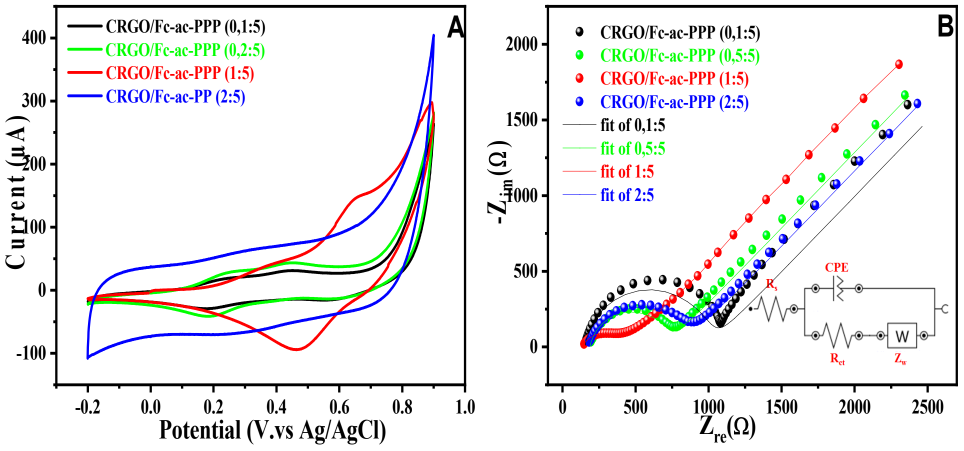


**Figure S4.** (A) CVs and (B) EIS of Au/CRGO/Fc-ac-PPP obtained with different mass ratio analysed in 10mM PBS solution pH=7 free of redox marker. CVs are obtained with scan rate 50mV/s and. EIS amplitude 10mV and frequency range from 100 KHz to 100 mHz.

*SI.2.3. Electron transfer kinetics determination from voltammetry*

The most popular method for the kinetic study is CV from which the peak-to-peak separation (ΔEp) between the oxidation and the reduction of the redox mediator was used to determine the heterogeneous electron transfer rate constant ($k_0$). For the estimation of $k_0$ value, we used the Nicholson method simplified by the work reported by Lavagnini et al [4]. The method of Nicholson is applicable to ΔEp < 220 mV, and is based on the following equation:

$$\psi = k^0 \sqrt{\frac{RT}{\pi nFD}}\, V^{0.5}$$

where $\psi$ is the dimensionless kinetic parameter calculated from ΔEp, R is the universal gas constant, T is the thermodynamic temperature, n is the number of electrons involved in the reaction, F is the Faraday constant, D is the diffusion coefficient of the electroactive species and ν is the scan rate. Through this mathematical manipulation, $k_0$ is taken directly from the slope of the graph of $\psi$ versus $\sqrt{\frac{RT}{\pi nFD}}\, V^{0.5}$. The kinetic parameter $\psi$ was calculated from ΔEp using the empirically determined working function by Lavagnini et al.

$$\psi = (-0.62880.0021X)/(1 - 0.017X)$$

where $X\ is\ \Delta Ep$ in mV .

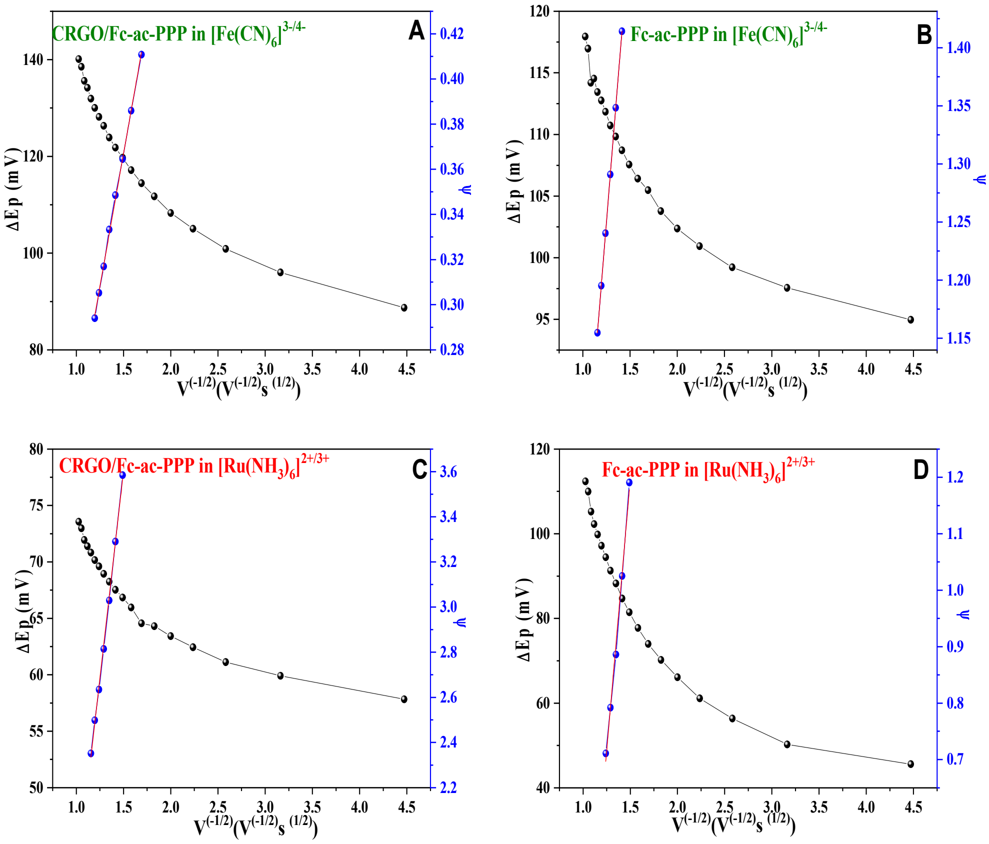


**Figure S5.** Relationship of $\Delta E_P$ and $\psi$ versus $v^{-1/2}$ of Au electrode modified with CRGO/Fc-ac-PPP (A, C) and Fc-ac-PPP (B,D) with $[Fe(CN)_6]^{3-/4-}$ and $[Ru(NH_3)_6]^{2+/3+}$ redox marker.

*SI.2.4 Analytical Performance Regarding DA, AA, UA*

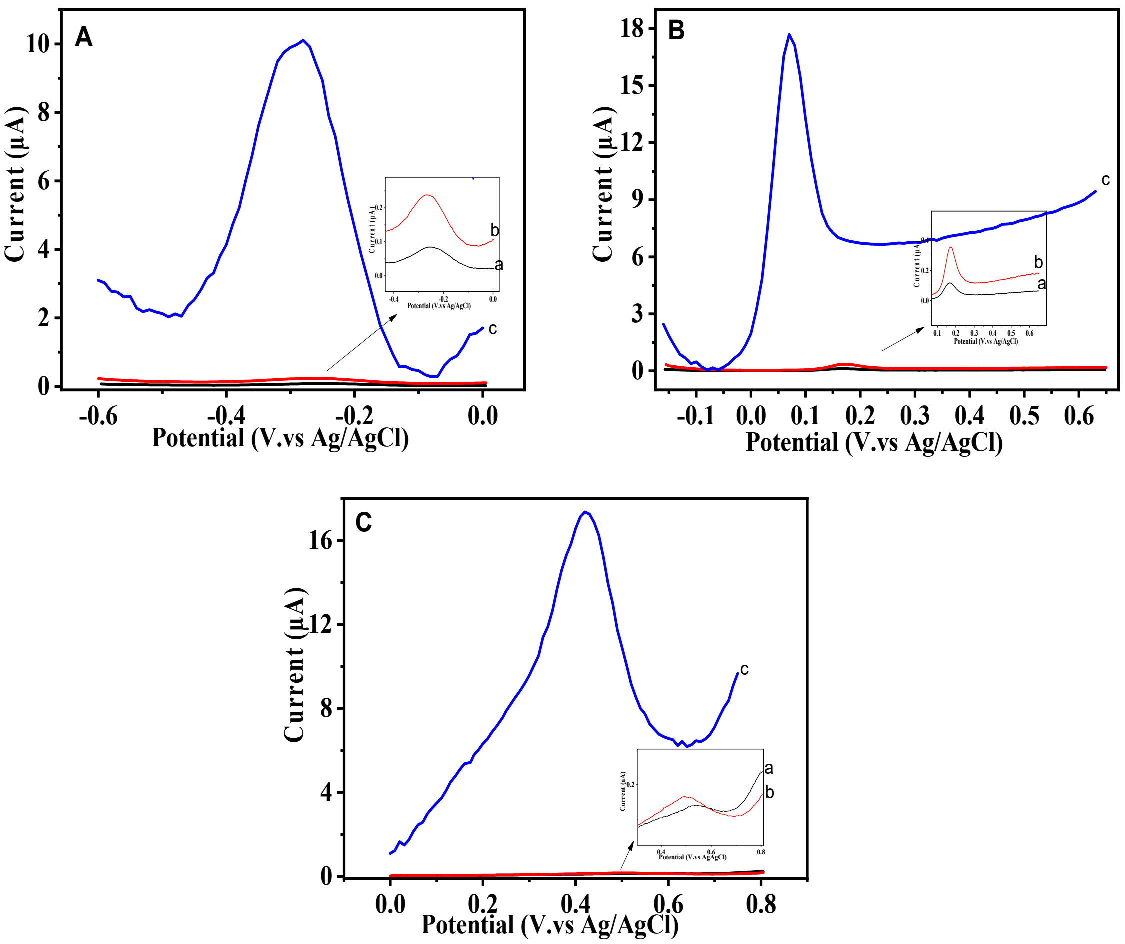


**Figure S6.** DPVs responses of (A) $10^{-4}$ M AA , (B) $10^{-5}$ M DA and (C) $10^{-4}$ M UA at (a) bare Au, (b) Au/Fc-ac-PPP and (c) Au/RGO/Fc-ac-PPP in 0,1M PBS (pH=7).


1. Blili, S.; Zaaboub, Z.; Maaref, H.; Said, A.H. Synthesis of a new p-conjugated redox oligomer: Electrochemical and optical investigation. *Journal of Molecular Structure.* **2017**,1128, 111-116.

2. Bizid, S.; Blili, S.; Mlika, R.; Said, A.H.; Youssoufi, H.K. Direct Electrochemical DNA Sensor based on a new redox oligomer modified with ferrocene and carboxylic acid: Application to the detection of Mycobacterium Tuberculosis mutant strain. *Analytica Chimica Acta*. **2017**, 994, 10-18.

3. Wang, S.; Han, Z.; Li,Y.; Peng, R.; Feng, B. Electrochemical sensor based on reduced graphene oxide and copper sulfide hollow nanospheres. *RSC Advances*. **2015**, 5, 107318-107325

4. Randviir, E.P. A cross examination of electron transfer rate constants for carbon screen-printed electrodes using Electrochemical Impedance Spectroscopy and Cyclic Voltammetry. *Electrochimica Acta*. **2018**, 286, 179-186.
